# Terminology in “Rural Community Knowledge of Stroke Warning Signs and Risk Factors”

**Published:** 2005-06-15

**Authors:** Michael W Day

**Affiliations:** Northwest MedStar, Spokane, Wash

To the Editor:

While I appreciate the effort of Blades et al in conducting the valuable research for "Rural Community Knowledge of Stroke Warning Signs and Risk Factors" (1), I believe that respondents to the survey do not necessarily represent rural communities. The authors cite county population densities as justification for using this classification; however, Cascade and Yellowstone counties contain two of the largest cities in the state of Montana. Statistically, the survey would have had to include a disproportionately large number of respondents who live either in the city's limits or its nearby suburbs. In addition, the authors did not note this as a limitation of the study. A survey using zip codes for small towns (less than 5000 inhabitants) more than 30 miles from a tertiary health care facility would be a more appropriate methodology in determining a true rural community.
